# Encapsulation of Black Rice Bran Extract in a Stable Nanoemulsion: Effects of Thermal Treatment, Storage Conditions, and In Vitro Digestion

**DOI:** 10.1021/acsomega.3c07060

**Published:** 2024-03-08

**Authors:** Mohamed N. Saleh, Mohamed Abdelbaset Salam, Esra Capanoglu

**Affiliations:** † Agricultural Research Center, Food Technology Research Institute, 3725004 Giza, Egypt; ‡ Department of Food Engineering, Faculty of Chemical and Metallurgical Engineering, Istanbul Technical University, Maslak, Istanbul 34469, Türkiye

## Abstract

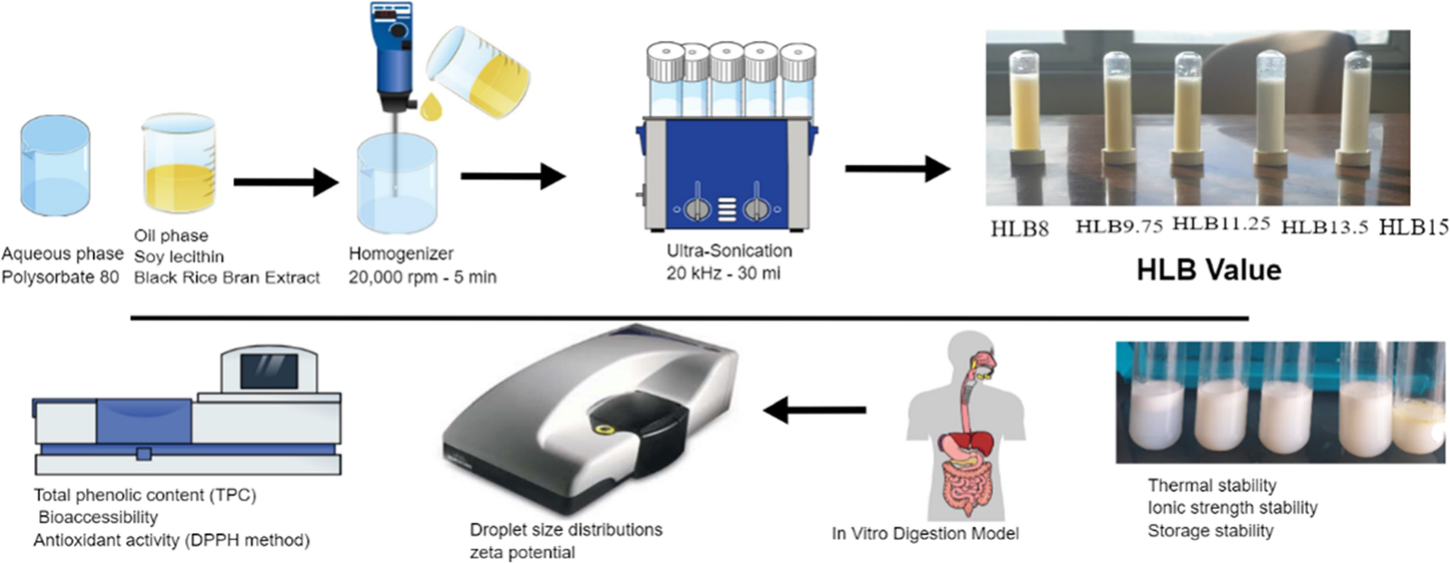

This study aimed to improve the dispersibility of phenolic compounds from black rice bran through the encapsulation process within nanoemulsion. The study focused on assessing the stability of the nanoemulsions, which were prepared using a combination of surfactants with distinct hydrophilic–lipophilic balance (HLB) values and sunflower oil under different thermal treatments and storage conditions. The study revealed a significant correlation between the mixed surfactant HLB value and the nanoemulsions properties, including average particle size, polydispersity index (PDI), and ζ-potential. Specifically, an increase in the HLB value was associated with a decrease in the initial average particle size. The encapsulated polyphenols exhibited remarkable stability over a storage period of up to 30 days at different temperatures with no significant changes observed in particle size or PDI. The study also investigated the impact of different ionic strengths (0.2, 0.5, and 1.00 mol L^–1^ NaCl) on the physical stability and antioxidant black rice bran extract nanoemulsion, and the results revealed that adding NaCl influenced the particle size and surface charge of the nanoemulsions. Total phenolic content and DPPH results demonstrated a significant impact of salt concentration on antioxidant properties, with varying trends observed among the HLB formulations. Furthermore, the behavior of the encapsulated extracts during digestion was examined, and their antioxidant activity was evaluated.

## 1 Introduction

Recently, there has been a growing interest in utilizing bioactive compounds from agricultural byproducts in the food and pharmaceutical industries.^1^ Rice bran is a significant component of brown rice and consists of outer layers, which are usually milled off during the production of white rice. Black rice bran contains several antioxidants, such as anthocyanin and phenolic acids, which effectively inhibit the formation and scavenging of free radicals that can cause cell damage.^2^

Nanoemulsions are dispersions of two liquids (water and oil) that are not typically compatible, which are stabilized by surfactant molecules forming a film at their interface. There are many advantages to using nanoemulsions, such as improved bioavailability, enhanced physical stability, improved solubilization of lipophilic drugs, and helped mask taste.^3^

The hydrophilic–lipophilic balance (HLB) is an index of solubilizing properties of surfactants, with scale ranges between 0 and 20.^4^ The surfactant systems with HLB values in the field of 3–6 usually produce w/o nanoemulsion, while the systems with HLB values in the area of 8–16 tend to produce o/w nanoemulsion. Furthermore, nanoemulsions stability depends on the critical role played by HLB.^5^

Polysorbate 80 (PS80) and soy lecithin (SL) are widely used emulsifiers with different molecular characteristics.^6^ SL has a long history of use as an emulsifier in the food industry, often paired with synthetic surfactants to reduce consumption.^7^ Furthermore, SL has an HLB value of 8.^8^

The effect of emulsifier type on lipid digestion and nutraceutical bioaccessibility can be investigated using the recently standardized INFOGEST digestion model, which allows for a comprehensive understanding of the intricate processes involved in the absorption and availability of bioactive compounds within the human body. Previous research utilizing various simulated gastrointestinal tract (GIT) models has already indicated that the choice of emulsifier can significantly influence these factors.^9−11^ In an effort to develop a stable nanoemulsion utilizing black rice bran extract (BRBE) for potential applications in the food industry, a comprehensive analysis was undertaken to assess the stability of the nanoemulsion under different thermal treatments and storage conditions. Furthermore, the behavior of the nanoemulsion was investigated during in vitro digestion to simulate physiological conditions.

## 2 Materials and Methods

### 2.1 Rice Bran Stabilization

The stabilization process of rice bran involved the microwaving of a black rice variety (*Oryza sativa* L.) using an LG microwave oven with 950 W output power for 3 min (around 78 °C) with manual mixing every minute after milling as proposed by Saleh et al.^12^ The black rice was sourced from the Rice Research and Training Center in Kafrelsheikh Governorate, Egypt, during the 2021 season.

### 2.2 Extraction of Phenolic Compounds

The extraction of antioxidants from black rice bran was conducted using a modified technique based on the method outlined.^13^ Five grams of rice bran powder was dissolved in 100 mL of 75% (v/v) aqueous ethanol. The mixture was subjected to sonication in a cooled ultrasonic bath to keep the temperature below 4 °C for 30 min, followed by centrifugation at 4 °C and 2700*g* for 10 min. The resulting supernatants were carefully collected. Subsequently, the solvent was eliminated by a rotary evaporator at 60 °C. Finally, the rice bran extracts were carefully stored in a brown bottle at −20 °C until further analysis.

### 2.3 Total Phenolic Content and Antioxidant Activity in BRBEs

The spectrophotometric method described by Waterhouse^14^ was used to determine the total phenolic content (TPC). 100 μL of BRBE or BRBE nanoemulsion (BRBE-NEe) was mixed with 7.9 mL of H_2_O, 500 μL of Folin-Ciocalteu reagent, and 1.5 mL of Νa_2_CO_3_ saturated solution. The absorbance of the mixture was measured after 2 h at 765 nm (UV2500UV–Vis, Shimadzu Co., Japan). The results were expressed as milligrams of gallic acid equivalents (mg GAE/100g extract). Furthermore, the radical scavenging capacity was evaluated using the DPPH method.^13^ 0.5 mL of BRBE or BRBE-NEe was mixed with 5 mL of a 0.1 mM DPPH solution and left to stand at room temperature for 30 min, before measuring the absorbance at 517 nm.^13^ The percentage inhibition was calculated using the following equation:
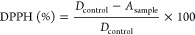
1where *D* is the absorbance of the control, and *A* is the absorbance of the sample at 517 nm.

### 2.4 HLB Calculation

To evaluate the impact of HLB values on the physicochemical characteristics of nanoemulsions, the HLB values were calculated through the following equation^15^
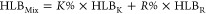
2where HLB_K_, HLB_R_, and HLB_Mix_ were the HLB values assigned to PS80, SL, and mixed surfactants, respectively. *K*% and *R*% represent the weight percentages of PS80 and SL in the mixed surfactants, respectively.

### 2.5 Fabrication of BRBE-NEe

The evaluated combinations of PS80 and SL, each possessing distinct HLB values of 15.0, 13.25, 11.5, 9.75, and 8.0, were prepared at the following ratios: 1:0, 3:1, 2:2, 1:3, and 0:1, respectively. A mixture of BRBE (0.1 wt %) and sunflower oil (7 wt %) with 3 wt % of emulsifier (SL) was stirred at 800 rpm for 2 h at room temperature. The lipid phase containing SL was incrementally added dropwise into the aqueous phase containing PS80 and homogenized (IKA, T18 digital ULTRA TURRAX) at 20,000 rpm for 5 min. The emulsion underwent additional processing through a 30 min ultrasonication at a frequency of 20 kHz in an ice bath.

### 2.6 Determination of Droplet Size Diameter, Polydispersity Index, and ζ-Potential

The determination of droplet size diameter, polydispersity index (PDI), and ζ-potential was carried out by using dynamic light scattering with the aid of a Zetasizer instrument (Mastersizer 2000, Malvern Instruments Ltd., Malvern, Worcestershire, U.K.). Before the particle size and PDI were measured at 25 °C, the samples were appropriately diluted 100-fold with distilled water.

### 2.7 Encapsulation Efficiency of Nanoemulsion Samples

The encapsulation efficiency (EE) of bioactive compounds was evaluated using a modified method introduced by Surassmo et al.^16^ Fifteen milliliters of the prepared nanoemulsion were filtered through a membrane cap, centrifuged (5000 rpm, 5 °C for 30 min), and the permeate was collected to measure TPC.

The EE of TPC was determined by the following equation:


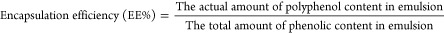
3

Actual polyphenol content in emulsion = TPC in emulsion – TPC in permeate.

### 2.8 Thermal Stability

Approximately 10 mL of BRBE-NEe was incubated in the water bath at 65 °C for 30 min or 100 °C for 10 min following the method reported by Li et al.^17^ Then cooling to room temperature, droplet size, PDI, ζ-potential, TPC, and antioxidant activity were measured.

### 2.9 Ionic Strength Stability

To evaluate the stability of BRBE-NEe under varying ionic strengths, solutions of NaCl with concentrations of 1, 0.5, and 0.2 mol L^–1^ were introduced into the system. Subsequently, measurements were taken for droplet size, PDI, ζ-potential, TPC, and antioxidant activity to assess any alterations resulting from the increase in the ionic strength.

### 2.10 Storage Stability

A nanoemulsion sample was stored under controlled temperatures of 4, 25, and 50 °C while maintained under dark conditions. Over 30 days, droplet size, PDI, and ζ-potential measurements were taken at 10 day intervals to monitor and analyze any potential variations in these parameters.

### 2.11 In Vitro Digestion Model

The in vitro digestion process was conducted in two stages, simulating GIT digestion following a standardized protocol.^10^ To summarize, each sample (10 mL) was combined with simulated gastric fluids containing pepsin (25,000 U/mL), HCl (1 M), and CaCl_2_ (0.3 M), resulting in a final pH of 3, then incubated in a shaking water bath at 37 °C for 30 min. Subsequently, simulated intestinal fluids, comprising pancreatin (5.0 mg/mL), bile salts (160 mM), CaCl_2_ (0.3 M), NaOH (1 M), phospholipids (1 mM), and phospholipase A2 (6.7 mg/mL stock solution), were added and incubated at 37 °C and pH 7 for 2 h.

The measurement of bioaccessibility followed the method described by Tan et al.^11^ After a 2 h digestion process in the small intestine, the “digesta” sample was collected. Subsequently, 15 mL of this sample were subjected to centrifugation (18,000 rpm, 4 °C) for 30 min, and the clear middle phase, referred to as the “micelle phase,″ was collected for analysis.

The bioaccessibility (BI) was calculated by the following equation:
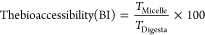
4

The TPC of samples was measured at the end of the digestion process, specifically in the micellar phase and digesta, referred to as *T*_Micelle_ and *T*_Digesta_, respectively.

### 2.12 Statistical Analysis

The data in this study were expressed as mean ± SD and analyzed using SPSS (Version 20.0). A one-way ANOVA was used to compare the results, and statistical significance was set at a *p* value of <0.05.

## 3 Results and Discussion

### 3.1 Effect of the HLB Value of the Mixed Surfactants

**Figure 1:**
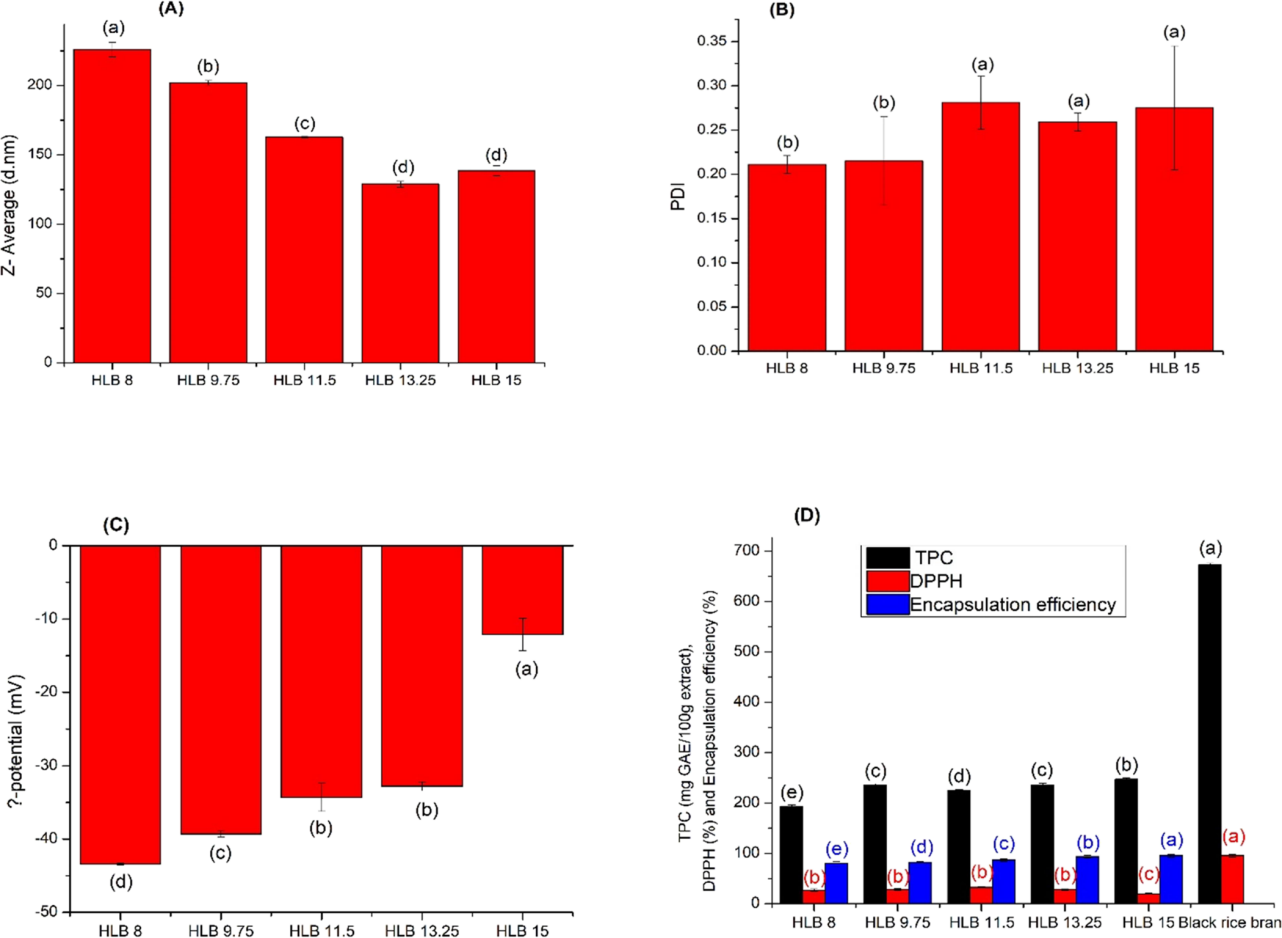
Particle size (A), PDI (B), ζ-potential (C), and EE, TPC, and antioxidant activity (D) of BRBE-NEe with a surfactant HLB (8–15). Error bars represent the standard deviation.

The influence of the HLB value of the mixed surfactants on the characteristics of BRBE-NEe was presented in Figure 1. Low molecular weight is the optimal emulsifier characteristic for forming small droplets.^18^ Despite SL having a molecular weight lower than that of PS80, the SL emulsion (HLB 8) was the only sample in the study with a larger droplet size than the others. The droplet sizes for HLB 15 and HLB 8 were 138.5 and 225.8 nm, respectively, as shown in Figure 1A. PS80 easily outperforms SL regarding diffusion rate, resulting in the oil droplets shrinking due to the rapid adsorption of PS80 molecules onto their surface.^19^ This could be because PS80 and SL have different HLBs. A high HLB value emulsifier can effectively stabilize an oil-in-water emulsion by forming droplets different from those of a low HLB value emulsifier. Thus, HLB 8 had a larger droplet size than those of the other samples. In addition, there is a difference between PS80 and SL as surfactants. PS80 has a single tail, while SL has two hydrocarbon tails. PS80 promotes fast interfacial adsorption, resulting in smaller droplets.^18^ Droplet size was further reduced by combining PS80 and SL. The droplet sizes of HLB 9.75, HLB 11.5, and HLB 13.25 were 162.7, 128.8, and 138.5 nm, respectively. These results aligned with the results of Lee et al.^20^

The PDI of a nanoemulsion indicates good homogeneity. The PDI of the nanoemulsions increases significantly as the HLB value increases. The nanoemulsions produced at HLB 8 to 15 showed PDI values of 0.211, 0.215, 0.281, 0.259, and 0.275, respectively, as presented in Figure 1B. Figure 1C presents the ζ-potential values formulated with varying PS80 and SL ratios. The ζ-potential for HLB 15 was −12.1 ± 2.2 mV, while the other samples had values around −32 mV or more. Notably, the combination of PS80 and SL in the black rice bran nanoemulsion formulation presented increasingly negative ζ-potential values of −39.3 to −32.8 mV as the SL ratio increased. In contrast, the SL emulsified nanocarrier without PS80 (HLB 8) had the lowest value.

Based on prior studies,^21,22^ the differences in ζ-potential values between nanocarriers emulsified with PS80 and SL can be attributed to their distinct surfactant properties. PS80 helps the stability of oil droplets using steric repulsion. On the other hand, SL, characterized as an amphiphilic surfactant, demonstrates negatively charged phospholipid head groups. This property enables the stabilization of the oil droplets through electrostatic repulsion. Consequently, the nanocarriers formulated with SL showed a higher electronegativity than those formulated with PS80.

The ζ-potential value of HLB 15, formulated with PS80 as the surfactant, exhibited a minor negative charge. This negative charge can be attributed to various factors, as anionic-free fatty acids and anionic impurities in the carrier oil contribute to the observed negativity. Furthermore, the oil–water interfaces can selectively adsorb hydroxyl ions from the surrounding water environment, potentially influencing the measured ζ-potential values.^19^

TPC, the EE, and antioxidant activity of the samples are presented in Figure 1D. The results demonstrated that BRBE recorded TPC reached about 672.5 mg GAE/100 g extract. The TPC of BRBE-NEe varied between 193.5 and 247.1 mg GAE/100 g extract for different HLB ratios. BRBE contains lipophilic antioxidants at a high level, together with a considerable amount of hydrophilic antioxidants, specifically anthocyanins.^23^ Adding a lipophilic emulsifier such as SL to the lipid phase improves the hydrophobic interactions and hydrogen bonding with the polyphenolic compounds, which are scarcely soluble in both the lipid and aqueous phases.^24^

The HLB 15 showed the highest TPC value of 247.1 mg GAE/100 g extract. After the emulsification process, the antioxidant activity in the nanoemulsions was decreased with a DPPH value of 19.1–32.6%. The type of emulsifier and any consequent changes in the HLB for stabilizing emulsions can significantly impact the behavior of antioxidants. This influence arises from the attractive or repulsive interactions between charged emulsion droplets and antioxidants, ultimately dictating where antioxidants are positioned within the emulsion.^25,26^ As demonstrated by Velasco et al., the activity of antioxidants such as gallic acid in a sunflower oil-in-water emulsion was closely linked to the ionic properties of the emulsifier (HLB) used.^27^

EE is a crucial characteristic, proving a therapeutic agent’s loading capacity in nanoemulsions and calculated using the TPC of the nanoemulsion. EE was significantly affected by different ratios of SL and PS80. The EE for all formulations ranged from 79.5 to 95.6%. The PS80 ratios increased the EE values. The trend is noticeable, especially regarding HLB 15, which exhibited the highest EE among the different HLB ratios, reaching 95.6%. In a study, encapsulating the polyphenolic extracts from grape marc in a nanoemulsion resulted in lower antioxidant activity of the encapsulated extracts than the unencapsulated compounds.^28^ Gaber Ahmed et al. also studied the process of nanoencapsulation for apple pomace phenolic extract using nanoemulsification. Interestingly, their findings indicated that the antioxidant activity of the phenolic extract was reduced when it was encapsulated, compared to the unencapsulated sample.^29^

### 3.2 Thermal Stability Study

The results in Table 1 show the impact of thermal treatment on the average particle size, PDI, ζ-potential, TPC, and antioxidant activity of BRBE-NEe. At a temperature of 65 °C, the average particle size varied between 229.6 and 338.5 nm. Notably, the droplet sizes of HLB 9.75, HLB 11.5, and HLB 15 at 65 °C were slightly larger than HLB 8. However, when exposed to a temperature of 100 °C, the average particle size was increased, particularly at higher concentrations of PS80, reaching 642.2 nm in HLB 15. This phenomenon can be attributed to the heat treatment temperature proximity to the nonionic surfactant phase inversion temperature. When the heat treatment temperature approaches this point, droplets tend to coalesce, leading to the instability of the nanoemulsions.^30^

**Table 1:** Z-Average, PDI, ζ-Potential, TPC, and Antioxidant Activity of BRBE-NEe under Thermal Treatment^a^

sample	treatment	*Z*-average (d.nm)	PDI	ζ-potential (mV)	TPC (mg GAE/100 g extract)	DPPH (% inhibition)
HLB 8.0	65 °C	229.6 ± 1.9^f^	0.200 ± 0.005^d^	–44.7 ± 0.5^e^	376.5 ± 1.8^b^	49.0 ± 3.4^a^
	100 °C	235.8 ± 0.6^f^	0.203 ± 0.011^d^	–47.0 ± 0.8^f^	490.6 ± 1.4^a^	43.0 ± 2.1^c^
HLB 9.75	65 °C	282.8 ± 1.3^d^	0.584 ± 0.241^ab^	–39.3 ± 0.2^c^	531.2 ± 1.7^a^	48.6 ± 1.5^a^
	100 °C	307.9 ± 3.1^c^	0.578 ± 0.080^ab^	–45.8 ± 0.8^ef^	548.7 ± 2.6^a^	45.7 ± 2.4^b^
HLB 11.5	65 °C	263.5 ± 2.1^e^	0.362 ± 0.017^c^	–39.9 ± 0.5^c^	516.4 ± 1.9^a^	47.2 ± 1.9^ab^
	100 °C	297.5 ± 2.1^c^	0.471 ± 0.054^bc^	–40.7 ± 1.9^c^	498.3 ± 2.9^a^	43.3 ± 3.4^c^
HLB 13.25	65 °C	234.0 ± 2.1^f^	0.267 ± 0.014^a^	–39.5 ± 0.4^c^	287.7 ± 2.2^c^	48.4 ± 1.6^a^
	100 °C	266.8 ± 3.9^e^	0.268 ± 0.006^d^	–42.1 ± 0.8^d^	191.0 ± 1.6^d^	50.8 ± 1.4^a^
HLB 15	65 °C	338.5 ± 1.7^b^	0.465 ± 0.08b^c^	–13.0 ± 0.4^a^	298.1 ± 1.4^c^	40.6 ± 2.4^d^
	100 °C	642.2 ± 2.9^a^	0.661 ± 0.076^a^	–19.8 ± 0.4^b^	324.2 ± 1.8^c^	39.3 ± 1.6^d^

a Values are means ± SD. Means having the different small case letter(s) within a column are significantly different at a *p* value of ≤0.05.

The stability and homogeneous droplet size distribution of an emulsion system are often indicated by a PDI value below 0.3.^31^ The PDI values of nanocarriers emulsified with HLB 9.75, HLB 11.5, and HLB 15 exhibited an increase exceeding 0.3 after being exposed to 65 and 100 °C, indicating an unstable emulsion system. Conversely, the PDI values of HLB 8 and 13.25 remained below 0.3 after thermal treatments, suggesting a more stable emulsion system. These observations can be attributed to the behavior of the head groups of PS80 under severe conditions. When exposed to such conditions, the head groups can become dehydrated, reducing steric forces responsible for preventing droplet coalescence and aggregation. Consequently, destabilization of the emulsion occurs.

In contrast, nanoemulsions containing SL demonstrated enhanced stability. One of the reasons for this enhanced stability is that the hydrophilic portion of the SL molecule remains hydrated even after thermal processing. The presence of hydrated hydrophilic groups helps maintain the overall stability of the nanoemulsion system, preventing droplet coalescence and aggregation.

The negative ζ-potential of nanoemulsions is another factor that may lead to their physical stability as it leads to increased repulsion between nanoparticles, resulting in more stable dispersions.^32^ Thermal processes can induce significant changes in the electrical charges present in nanoemulsions, as indicated in Table 1. The SL nanoemulsion exhibited good thermal stability and a high negative ζ-potential (Table 1). This increased negative charge contributes to the enhanced stability of the nanoemulsion system. The results showed that even after heat treatment, a substantial electrostatic repulsion could be maintained between the droplets of the SL nanoemulsion. A general trend is that increasing temperature tends to decrease the ζ-potential as it reduces the viscosity and dielectric constant of water, increases the dissociation of acidic groups on particles and surfactants, and decreases the solubility and interfacial tension of the oil. However, this trend may not apply to all systems as there may be exceptions or nonlinear effects due to specific interactions or phase transitions.^33^

Table 1 presents the TPC and antioxidant activity results of nanoemulsions with different HLB values under thermal treatment. The nanoemulsions were exposed to temperatures of 65 and 100 °C. The results show that the nanoemulsions with the HLB values (9.75, 11.5, and 13.25) had significantly higher TPC values than those with HLB values (8.0 and 15.0) at 65 and 100 °C. The antioxidant activity of the nanoemulsions was assessed using the DPPH assay. The nanoemulsion with HLB 13.25 had an inhibition value of 50.8% at 100 °C, with the highest increasing trend in antioxidant activity. These findings demonstrate that both the HLB value and the temperature significantly impact the properties of BRBE-NEe. The increase in TPC and DPPH values could be due to the fact that higher temperatures may promote the release and availability of bioactive compounds from the nanoemulsion. However, it is essential to note that the optimal temperature for achieving the highest DPPH inhibition may vary depending on the sample.^34^ Also, the surfactants can affect the physical location of antioxidants in emulsions by solubilizing lipid-soluble antioxidants in the aqueous phase.^35^

According to a study by Losada-Barreiro et al.,^36^ the HLB value of nonionic surfactants such as Tween 20, 40, 80, and Span 20, as well as their proportion within emulsions, may facilitate the inclusion of hydrophilic antioxidants within the interfacial regions of the emulsion. Furthermore, Velderrain-Rodrguez et al. have indicated that in the context of emulsions, employing high HLB surfactants such as Tweens at a surfactant volume fraction of 0.04 leads to the encapsulation of more than 90% of gallic acid within the interfacial region. This positioning of antioxidants could hold importance in enhancing the stability of emulsions.^37^

### 3.3 Ionic Strength Stability Study

The effect of ionic strength (0.2, 0.5, and 1.00 mol L^–1^) on the physical stability of BRBE-NEe was investigated (Table 2). The average particle size of HLB 8 increased to 422.7 nm with increasing NaCl content to 1.0 mol L^–1^. It is known that exceeding the critical salinity leads to an insufficient electrostatic repulsion, allowing attractive interactions like hydrophobic and van der Waals forces to prevail.^38^ When PS80 and SL were combined, smaller droplets were achieved for HLB 9.75 and 11.25 at NaCl concentrations of 0.2 and 0.5 mol L^–1^, measuring approximately 187.3, 189.2, 173.5, and 155.1 nm, respectively. However, as the NaCl concentration increased to 1.0 mol L^–1^, a more substantial increase in droplet size was observed. On the contrary, the nanoemulsions containing HLB 13 remained remarkably stable even with increasing salt concentrations, as evidenced by no changes in size.

**Table 2:** Effect of Different Salt Concentrations on *Z*-Average, ζ-Potential, TPC, and Antioxidant Activity of BRBE-NEe^a^

	*Z*-average (d.nm)			ζ-potential (mV)			TPC (mg GAE/100 g extract)			DPPH (% Inhibition)		
sample	NaCl 0.2 mol L^–1^	NaCl 0.5 mol L^–1^	NaCl 1.0 mol L^–1^	NaCl 0.2 mol L^–1^	NaCl 0.5 mol L^–1^	NaCl 1.0 mol L^–1^	NaCl 0.2 mol L^–1^	NaCl 0.5 mol L^–1^	NaCl 1.0 mol L^–1^	NaCl 0.2 mol L^–1^	NaCl 0.5 mol L^–1^	NaCl 1.0 mol L^–1^
HLB 8.0	284.3 ± 13.6^aB^	447.3 ± 20.5^aA^	422.7 ± 18.9^aA^	–45.0 ± 0.9^cB^	–44.4 ± 1.3^cAB^	–42.5 ± 0.6^cA^	541.4 ± 2.1^bA^	186.5 ± 1.7^eB^	23.4 ± 1.5^eC^	45.5 ± 1.9^cB^	52.4 ± 2.5^aA^	22.9 ± 1.7^cC^
HLB 9.75	187.3 ± 3.7^bB^	189.2 ± 5.1^bB^	220.5 ± 29.7^bA^	–34.7 ± 1.0^bB^	–33.8 ± 1.1^bB^	–32.1 ± 0.7^bA^	638.4 ± 2.5^aA^	198.7 ± 2.6^dB^	89.8 ± 1.2^cC^	49.7 ± 1.7^aA^	43.3 ± 1.5^cB^	38.6 ± 1.6^aC^
HLB 11.5	173.5 ± 5.6^bB^	155.1 ± 4.7^bcC^	238.2 ± 16.7^bA^	–34.8 ± 1.0^bB^	–33.1 ± 0.6^bB^	–28.1 ± 1.2^bA^	328.1 ± 1.9^dB^	746.3 ± 2.5^aA^	134.5 ± 1.2^aC^	46.3 ± 1.6^bA^	44.3 ± 1.3^cB^	36.2 ± 1.2^bC^
HLB 13. 25	125.7 ± 3.9^cA^	124.6 ± 4.1^cA^	125.3 ± 1.9^cA^	–33.9 ± 0.8^bB^	–33.1 ± 0.6^bB^	–30.4 ± 0.7^bA^	466.5 ± 2.4^cA^	225.1 ± 1.2^bB^	71.5 ± 1.2^dC^	50.2 ± 1.3^aA^	47.4 ± 1.9^bB^	38.9 ± 1.5^aC^
HLB 15	270.3 ± 26.85^aA^	208.7 ± 41.8^bB^	264.8 ± 13.0^bA^	–14.5 ± 1.4^aA^	–14.9 ± 0.4^aA^	–14.1 ± 0.3^aA^	262.5 ± 2.6^eA^	208.2 ± 1.9^cB^	109.8 ± 1.5^bC^	47.5 ± 2.3^bA^	12.9 ± 1.4^dB^	11.7 ± 1.9^dB^

a Values are means ± SD. Means having the different capital case letter(s) within a row (for each assay individually) are significantly different at *p* < 0.05. Means having the different small case letter(s) within a column are significantly different at *p* < 0.05.

The ζ-potential data showed that all samples containing SL with PS80 increased in negative charge value as the salt concentration increased. The increase in negative charge can be attributed to the aggregation of Na^+^ around the droplet surfaces, leading to electrostatic screening effects and a reduction in net charge.^39^ Interestingly, the ζ-potential values of HLB 15 remained relatively stable, averaging at −14.3 mV. This finding showed that PS80 facilitated the formation of more permanent layers on the droplet surfaces, contributing to their stability. The observed results are primarily attributed to the stabilizing impact of steric repulsion, rather than electrostatic interactions, in these nanoemulsions.^22^

Table 2 illustrates the impact of different salt concentrations on the TPC and DPPH of BRBE-NEe. The results from this study demonstrate that the TPC and DPPH of BRBE-NEe were significantly affected by different salt concentrations. All HLBs showed a decrease in the level of TPC with increasing NaCl concentrations. On the other hand, the TPC of HLB 11.5 increased with an increase in salt concentration up to 0.5 mol L^–1^. However, there was a decrease at 1.0 mol L^–1^.

Furthermore, the NaCl concentration can influence DPPH values. For each HLB value, the DPPH assay results showed that the antioxidant activity of BRBE-NEe decreased with increasing salt concentrations. When the HLB 15 data are compared, the DPPH values decrease from NaCl 0.2 to NaCl 1.0 mol L^–1^. This indicates that higher NaCl concentrations may affect the antioxidant activity of the extracts. This could be because the phenolic compounds in BRBE-NEe become less available for scavenging free radicals as the salt concentration increases. Overall, this study demonstrates that salt concentrations significantly affect the antioxidant activity and TPC of BRBE-NEe. Increasing NaCl concentration can increase the ionic strength and decrease the electrostatic repulsion between the nanoemulsion droplets, leading to droplet aggregation or coalescence. This can reduce the surface area and exposure of phenolic compounds to DPPH radicals, resulting in lower antioxidant activity.^40^

Furthermore, increasing NaCl concentration can change the pH and redox potential of the nanoemulsion system, which can affect the stability and reactivity of phenolic compounds and DPPH radicals. This can influence the rate and extent of DPPH radical scavenging, resulting in lower or higher antioxidant activity depending on the pH and redox sensitivity of phenolic compounds.^41^ Therefore, TPC does not always correlate with antioxidant activity, as other factors can affect the antioxidant potential besides the amount of phenolic compounds.^42^ However, the exact mechanism of action needs further exploration.

### 3.4 Effect of Storage Temperature on the Stability of BRBE-NEe

The stability of the nanoemulsion can be influenced by adjusting the physical characteristics of the oil, water, and surfactant, which can, in turn, be affected by temperature.^43^ To investigate the stability of BRBE-NEe, measurements of the average particle size, PDI, and ζ (zeta)-potential value were conducted periodically over 30 days. The measurements were taken every 10 days, and the results are presented in Table 3.

**Table 3:** *Z*-Average Size, PDI, and ζ-Potential of the BRBE-NEe Stored at Various Temperatures^a^

HLB 8				
	0 days	10 days	20 days	30 days
*Z*-average (d.nm)				
4 °C		223.4 ± 3.1^aA^	192.1 ± 2.6^bB^	224.0 ± 2.0^aA^
25 °C	225.8 ± 5.2^A^	215.5 ± 3.1^aB^	216.0 ± 3.2^aB^	226.7 ± 2.8^aA^
50 °C		228.3 ± 11.7^aA^	215.8 ± 4.9^aA^	228.5 ± 5.3^aA^
PDI				
4 °C		0.157 ± 0.01^bA^	0.141 ± 0.01^bA^	0.138 ± 0.01^bA^
25 °C	0.211 ± 0.01^A^	0.190 ± 0.01a^aA^	0.211 ± 0.0^aA^	0.216 ± 0.01^aA^
50 °C		0.161 ± 0.01b^bB^	0.148 ± 0.01^bB^	0.133 ± 0.01^bC^
ζ-potential (mV)				
4 °C		–44.4 ± 0.5^aA^	–44.9 ± 1.4^aA^	–45.2 ± 1.5^aA^
25 °C	–43.4 ± 0.1^A^	–46.8 ± 0.6^aA^	–45.6 ± 0.7^aA^	–46.1 ± 1.0^aA^
50 °C		–47.1 ± 0.9^aA^	–46.5 ± 0.8^aA^	–49.3 ± 0.5^aA^

HLB 9.75				
	0 days	10 days	20 days	30 days
*Z*-average (d.nm)				
4 °C		201.7 ± 8.1^aA^	168.9 ± 4.4^bB^	180.9 ± 7.4^aC^
25 °C	201.9 ± 3.7^A^	168.9 ± 3.8^bD^	192.1 ± 4.9^aB^	182.9 ± 6.4^aC^
50 °C		197.5 ± 11.8^aA^	168.1 ± 3.1^bB^	161.5 ± 3.2^bB^
PDI				
4 °C		0.240 ± 0.01^aA^	0.241 ± 0.01^aA^	0.236 ± 0.02^aA^
25 °C	0.215 ± 0.05^A^	0.267 ± 0.08^aA^	0.249 ± 0.01^aA^	0.272 ± 0.01^aA^
50 °C		0.279 ± 0.10^aA^	0.244 ± 0.02^aA^	0.228 ± 0.017^aA^
ζ-potential (mV)				
4 °C		–40.5 ± 0.9^bA^	–40.9 ± 0.5^bA^	–42.8 ± 0.8^aA^
25 °C	–39.3 ± 0.4^A^	–40.9 ± 0.4^bA^	–43.1 ± 0.6^abA^	45.9 ± 0.4^abA^
50 °C		–45.4 ± 0.5^aA^	–44.1 ± 0.5^aA^	–47.9 ± 0.9^aA^

HLB 11.5				
	0 days	10 days	20 days	30 days
particle size (nm)				
4 °C		156.7 ± 2.9^aA^	142.2 ± 1.2^bB^	147.9 ± 5.3^aB^
25 °C	162.7 ± 2.6^A^	152.8 ± 4.2^aB^	159.3 ± 3.4^aAB^	162.4 ± 4.8^aA^
50 °C		137.6 ± 3.2^bB^	136.9 ± 2.8^cB^	137.3 ± 2.2^aB^
PDI				
4 °C		0.249 ± 0.01^aA^	0.227 ± 0.01^aB^	0.231 ± 0.01^aB^
25 °C	0.281 ± 0.03^A^	0.250 ± 0.01^aA^	0.203 ± 0.02^aB^	0.263 ± 0.01^aA^
50 °C		0.224 ± 0.07^aA^	0.237 ± 0.01^aA^	0.203 ± 0.01^aB^
ζ-potential (mV)				
4 °C		–40.5 ± 0.7^aA^	–40.7 ± 0.8^aA^	–40.8 ± 0.6^bA^
25 °C	–34.3 ± 1.9^B^	–37.5 ± 0.3^bA^	–37.4 ± 0.4^abA^	–38.9 ± 0.3^bA^
50 °C		–38.2 ± 0.6^abB^	–36.9 ± 0.9^bB^	–46.5 ± 0.6^aA^

HLB 13.25				
	0 days	10 days	20 days	30 days
*Z*-average (d.nm)				
4 °C		129.8 ± 2.8^aA^	126.4 ± 3.6^aA^	123.8 ± 4.5^bB^
25 °C	128.8 ± 2.1^A^	127.1 ± 1.8^aA^	127.4 ± 3.3^aA^	126.6 ± 2.9^aA^
50 °C		126.3 ± 3.5^aA^	124.5 ± 3.1^aAB^	123.7 ± 3.5^bB^
PDI				
4 °C		0.242 ± 0.01^bA^	0.257 ± 0.01^aA^	0.245 ± 0.01^aA^
25 °C	0.259 ± 0.01^A^	0.250 ± 0.01^aA^	0.236 ± 0.01^bB^	0.231 ± 0.01^bB^
50 °C		0.242 ± 0.01^bB^	0.257 ± 0.01^aA^	0.245 ± 0.01^aB^
ζ-potential (mV)				
4 °C		–32.4 ± 0.7^cB^	–34.8 ± 0.9^aAB^	–36.7 ± 0.6^bA^
25 °C	–32.8 ± 0.6^B^	–43.2 ± 0.6^aA^	–38.8 ± 0.7^aB^	–40.1 ± 0.4^aAB^
50 °C		–36.4 ± 0.5^bA^	–36.9 ± 0.5^aA^	–38.2 ± 0.8^aA^

HLB 15				
	0 days	10 days	20 days	30 days
*Z*-average (d.nm)				
4 °C		136.7 ± 3. 4^bA^	137.9 ± 5.3^bA^	138.0 ± 4.6^bA^
25 °C	138.5 ± 3.6^A^	128.2 ± 4.7^cB^	122.7 ± 2.3^cC^	120.7 ± 5.5^cC^
50 °C		153.9 ± 1.1^aC^	166.5 ± 5.2^aB^	209.6 ± 1.5^aA^
PDI				
4 °C		0.279 ± 0.03^bB^	0.288 ± 0.01^bB^	0.337 ± 0.02^bA^
25 °C	0.275 ± 0.01^B^	0.410 ± 0.01^aA^	0.417 ± 0.01^aA^	0.429 ± 0.08^aA^
50 °C		0.469 ± 0.11^aA^	0.486 ± 0.01^aA^	0.479 ± 0.03^aA^
ζ-potential (mV)				
4 °C		–19.2 ± 0.2^aA^	–18.8 ± 0.8^aA^	–19.60 ± 0.6^bA^
25 °C	–12.1 ± 2.2^B^	–20.5 ± 0.3^aA^	–21.9 ± 0.9^aA^	–21.30 ± 0.8^bA^
50 °C		–21.1 ± 0.7^aA^	–21.6 ± 0.3^aA^	–25.10 ± 095^aA^

a Values are means ± SD. Means having the different capital case letter(s) within a row are significantly different at *p* < 0.05. Means having the different small case letter(s) within a column are significantly different at *p* < 0.05.

According to the findings in Table 3, it was observed that the sample with an initial average droplet size of 225.8 nm (HLB 8) exhibited stability throughout the storage period at all tested temperatures, showing no significant variations in the average particle size. On the other hand, the sample with a smaller initial average droplet size (HLB 15) demonstrated stability when stored at temperatures of 4 and 25 °C. However, when stored at 50 °C for 30 days, an increase in the average particle size was observed. The observed increase in droplet size during storage can be attributed to the Ostwald ripening process, which is responsible for the destabilization of nanoemulsions.^44^ Moreover, at high temperatures, the Brownian motion of dispersed droplets resulted in coalescence or flocculation, increasing droplet size.^45^

During the storage period, samples with HLB of 9.75, 11.5, and 15 decreased particle size, regardless of the storage temperature. This phenomenon has also been observed by Akhoond Zardini et al.^32^ The reason for this is that the surfactant was given enough time to spread across the surfaces of the droplets and effectively coat them for storage.

The PDI values for the samples in Table 3 were generally low, indicating that it was monodisperse. Other researchers have observed similar results when encapsulating carotenoids in oil cores.^46^

Table 3 presents the ζ-potential data, indicating no significant alterations in the sample charge when stored at 4 and 25 °C. However, at 50 °C, the ζ-potential showed a more negative value toward the end of the storage period. The ζ-potential values for HLB 8 remained relatively stable throughout the storage period at different temperatures (4, 25, and 50 °C). The values ranged between approximately −44.4 and −49.3 mV, indicating that the nanoemulsion maintained its stability over time. The ζ-potential values for HLB 11.5 demonstrated a slight decrease over time at 4 and 25 °C. However, at 50 °C, there was a significant decrease in the ζ-potential values; while at HLB 13.25, the ζ-potential values remained relatively stable. The ζ-potential values for HLB 15 showed a slight decrease over time at 4 and 25 °C. At 50 °C, there was a more noticeable decrease in the ζ-potential values. This increase in negative charge can be attributed to the higher levels of free fatty acids present. Nevertheless, this effect does not significantly impact the overall stability of the sample. Moreover, the stability of the droplets can be attributed to multiple factors, including their small particle size, highly negative ζ-potential (exceeding −40 mV), and the long-chain triglycerides in the sunflower oil. Collectively, these factors contribute to the effective stabilization of the droplets, resulting in the delay of the Oswald ripening process.^47^

### 3.5 Effect of Storage Temperature on the TPC and Total Antioxidant Activity of BRBE-NEe

Table 4 presents the total antioxidant activity (DPPH %) and TPC of BRBE-NEe stored at varying temperatures over time. The samples were stored for 0, 10, 20, and 30 days at 25, 4, and 50 °C. TPC values for HLB 8 increased over time at all storage temperatures. The storage temperature played a significant role, with different treatments showing a peak phenolic content at specific temperatures. HLB 15 demonstrated the highest TPC after 20 days at 25 °C, while HLB 8 exhibited the highest values after 10 days at 4 °C. Moreover, the storage time interval influenced the TPC, with varying optimal time points for each sample. During the 30 days, there is an initial increase in TPC in HLB 9.75, HLB 11.5, and HLB 13.25 at 25 and 50 °C. However, TPC was observed to decrease after 20 and 30 days at both temperatures. The decrease in TPC at higher temperatures (50 °C) may be more pronounced, suggesting that the temperature plays a role in the stability or degradation of phenolic compounds in these emulsions. These findings indicate that the HLB value, storage temperature, and time interval are critical factors affecting the stability and concentration of phenolic compounds in BRBE-NEe.

**Table 4:** TPC and DPPH Values of BRBE-NEe Stored at Different Temperatures^a^

TPC (mg GAE/100 g extract)												
	25 °C				4 °C				50 °C			
	0 days	10 days	20 days	30 days	0 days	10 days	20 days	30 days	0 days	10 days	20 days	30 days
HLB8	193.5 ± 2.5^dD^	720.8 ± 1.6^cA^	533.2 ± 7.6^cB^	353.1 ± 17.1^dC^	193.5 ± 2.5^dD^	1761.2 ± 5.9^aA^	807.9 ± 4.3^dB^	239.37 ± 4.4^dC^	193.5 ± 2.5^dC^	505.5 ± 3.9^eB^	720.73 ± 3.6^bA^	507.67 ± 3.9^dB^
HLB9.75	235.8 ± 2.4^bC^	1567.2 ± 1.8^aA^	436.2 ± 36.6^dB^	374.1 ± 4.3^dB^	235.8 ± 2.4^bD^	863.84 ± 5.1^dA^	738.2 ± 4.8^eB^	357.44 ± 7.9^cD^	235.8 ± 2.4^bC^	1226.37 ± 5.1^aA^	627.99 ± 4.5^cdB^	671.56 ± 2.3^cB^
HLB11.5	224.6 ± 2.0^cD^	1249.5 ± 1.7^bA^	1101.6 ± 38.5^bB^	777.9 ± 24.7^cC^	224.6 ± 2.0^cD^	991.8 ± 4.9^cB^	1051.5 ± 10.8^aA^	473.9 ± 3.5^bC^	224.6 ± 2.0^cD^	1028.81 ± 3.6^bB^	581.36 ± 3.2^dC^	1241.36 ± 2.4^bA^
HLB13.25	235.6 ± 3.5^bC^	1190.22 ± 1.16^bA^	1084.6 ± 91.5^bA^	952.6 ± 23.3^aB^	235.6 ± 3.5^bD^	511.1 ± 3.1^eC^	976.5 ± 4.7^cA^	692.7 ± 4.8^aB^	235.6 ± 3.5^bD^	932.0 ± 2.7^cB^	735.80 ± 4.3^bC^	1547.49 ± 2.6^aA^
HLB15	247.1 ± 2.7^aD^	443.8 ± 1. 5^dC^	1277.8 ± 34.3^aA^	870.8 ± 31.5^bB^	247.1 ± 2.7^aD^	1172.7 ± 4.7^bA^	1007.8 ± 2.7^bB^	316.4 ± 2.7^cC^	247.1 ± 2.7^aD^	653.51 ± 3.6^dB^	932.79 ± 4.0^aA^	446.84 ± 2.89^eC^

Total Antioxidant Activity-DPPH (% Inhibition)												
	25 °C				4 °C				50 °C			
	0 days	10 days	20 days	30 days	0 days	10 days	20 days	30 days	0 days	10 days	20 days	30 days
HLB 8	27.1 ± 2.2^bC^	37.7 ± 1.9^aB^	46.5 ± 1.2^bA^	49.4 ± 1.17^aA^	27.1 ± 2.2^bB^	20.0 ± 1.5^cC^	38.0 ± 1.5^aA^	26.8 ± 1.3^cB^	27.1 ± 2.2^bD^	46.8 ± 1.0^bA^	40.6 ± 1.5^bB^	37.9 ± 1.7^cC^
HLB9.75	28.2 ± 1.8^bD^	37.61 ± 2.4^aC^	62.4 ± 2.2^aA^	48.09 ± 1.5^aB^	28.2 ± 1.8^bA^	23.6 ± 1.7^bcB^	22.2 ± 1.9^cB^	21.5 ± 2.0^dB^	28.2 ± 1.8^bD^	59.5 ± 2.3^aA^	41.9 ± 1.8^bB^	37.2 ± 1.7^cC^
HLB11.5	32.6 ± 1.5^aB^	29.80 ± 2.9^bC^	39.1 ± 2.5^cA^	42.0 ± 1.6^bA^	32.6 ± 1.5^aB^	25.8 ± 1.9^bC^	36.1 ± 1.8^aA^	31.8 ± 2.1^bB^	32.6 ± 1.5^aC^	57.5 ± 2.1^aA^	41.0 ± 1.3^bB^	39.9 ± 2.1^bB^
HLB13.25	27.9 ± 1.7^bD^	30.76 ± 1.1^bC^	45.7 ± 1.6^bA^	39.1 ± 1.5^cB^	27.9 ± 1.7^bB^	22.5 ± 1.3^bcC^	32.1 ± 1.8^bA^	22.8 ± 1.4^dC^	27.9 ± 1.7^bC^	58.0 ± 2.7^aA^	58.2 ± 2.46^aA^	48.4 ± 2.6^aB^
HLB15	19.1 ± 2.3^cD^	22.5 ± 2.9^cC^	39.2 ± 1.4^cB^	43.1 ± 1.6^bA^	19.1 ± 2.3^cC^	30.6 ± 1.8^aB^	30.1 ± 1.1^bB^	36.9 ± 1.1^aA^	19.1 ± 2.3^cC^	60.1 ± 2.6^aA^	28.6 ± 1.8^cB^	26.9 ± 1.6^dB^

a Values are means ± SD. Means having the different capital case letter(s) within a row (for each temperature individually) are significantly different at *p* < 0.05. Means having the different small case letter(s) within a column are significantly different at *p* < 0.05.

The total antioxidant activity, measured by DPPH (% inhibition), varied among the different HLB treatments and storage temperatures. The study investigated the influence of the storage temperature and time on DPPH values, which reflect the antioxidant capacity of different HLB values in a sample. The findings demonstrated that the DPPH values were affected by both factors. Storage at 25 °C increased DPPH values over time for all HLB values, with HLB 8, HLB 11.5, and HLB 15 displaying the highest values after 30 days. At 4 °C, HLB 11.5 initially exhibited the highest DPPH level, but HLB 9.75 and HLB 13.25 surpassed it after 10 and 20 days, respectively. HLB 8 demonstrated the highest DPPH value after 30 days. When stored at 50 °C, HLB 8 initially had the highest DPPH value, while HLB 11.5 showed the highest values after 10 and 20 days and HLB 9.75 displayed the highest DPPH level after 30 days.

It can be concluded that the storage duration and temperature significantly influenced the antioxidant capacity, with lower temperatures generally associated with higher antioxidant activity. Choulitoudi et al. investigated the impact of storage temperature (5 to 40 °C) on emulsions containing *Satureja thymbra* extract. They found that the phenolic content in these emulsions decreased during storage, and the rate of decline depended on the temperature at which they were stored.^48^ On the other hand, Di Mattiaet et al. observed similar trends in emulsions with different phenolic compounds. Emulsions containing quercetin showed increased antioxidant activity after 10 day storage, while those with catechin exhibited an initial rise in antiradical capacity followed by a subsequent decrease.^49^

In addition, the nature of the emulsifier used to stabilize the emulsions may have a remarkable effect on the action of antioxidants due to the attractive or repulsive interactions between charged emulsion droplets and antioxidants.^25^ But, overall, there was no clear trend between the antioxidant activity and storage duration and temperature. This suggested that other factors were also important in determining the efficacy of phenolics in the nanoemulsions. For instance, there might be differences in the chemical reactivity of different phenolic extracts that impacted their ability to inhibit oxidation.^50^

### 3.6 Effects of In Vitro Digestion

The bioaccesibility of nanoemulsified BRBE was evaluated (Table 5), and the results demonstrated a remarkable improvement in the bioaccessible amount of TPC achieved through nanoemulsification. Upon undergoing in vitro digestion, a notable increase in TPC was observed across all samples. The bioaccessibility values ranged from 37.3 to 76.3%.

**Table 5:** TPC and Bioaccessibility of BRBE-NEe^a^

sample	undigested nanoemulsion		after in vitro digestion process				bioaccessibility %
	TPC	DPPH	TPC		DPPH		
			digesta	micelle	digesta	micelle	
HLB8	193.5 ± 2.5^d^	27.1 ± 2.2^b^	1227.0 ± 3.8^b^	457.9 ± 1.8^d^	35.9 ± 3.6^a^	56.5 ± 3.5^b^	37.3 ± 1.5^e^
HLB9.75	235.8 ± 2.4^b^	28.2 ± 1.8^b^	1089.5 ± 3.8^c^	461.3 ± 2.8^d^	30.8 ± 2.1^bc^	62.7 ± 2.9^a^	42.3 ± 0.8^d^
HLB11.5	224.6 ± 2.0^c^	32.6 ± 1.5^a^	1093.0 ± 2.9^c^	557.9 ± 2.4^c^	29.7 ± 3.9^c^	61.7 ± 2.9^a^	46.9 ± 1.4^c^
HLB13.25	235.6 ± 3.5^b^	27.9 ± 1.7^b^	1361.9 ± 3.8^a^	829.2 ± 3.7^b^	26.8 ± 2.0^d^	57.4 ± 3.3^b^	60.9 ± 0.9^b^
HLB15	247.1 ± 2.7^a^	29.1 ± 2.3^b^	1271.9 ± 2.9^b^	970.8 ± 3.4^a^	32.5 ± 1.9^b^	57.8 ± 3.3^b^	76.3 ± 21.6^a^

a Values are means ± SD. Means having the different small case letter(s) within a column are significantly different at *p* < 0.05.

The results revealed a correlation between HLB levels and the bioaccessibility of phenolic compounds, with higher HLB values generally associated with increased bioaccessibility. The sample with the lowest HLB exhibited the lowest bioaccessibility at 37.3%. This can be attributed to the limited ability of SL to generate small oil droplets during homogenization, resulting in slower lipid digestion and reduced bioaccessibility.^11,51^ In contrast, using small molecule surfactants such as PS80 facilitated the formation of small and stable droplets, thereby promoting efficient lipid digestion and increasing bioaccessibility.^11^ In another study, researchers have similarly observed an increase in the TPC value of the emulsions in tomato pomace after exposure to in vitro digestion.^52^

The health benefits of nutraceuticals are frequently linked to their antioxidant properties. Hence, evaluating the antioxidant activity of BRBE-NEe following in vitro digestion was important. Table 5 presents the different HLB values related to the HLB of the nanoemulsion samples. The data showed that the highest DPPH inhibition was found in the sample with an HLB of 9.75, with an average of 62.7%, followed by samples with HLBs of 11.5 and 13.25, which had average DPPH inhibition values of 61.7 and 57.4%, respectively. The lowest DPPH inhibition was found in the sample with an HLB of 8, with an average of 56.5%. The results suggest that the higher the HLB value, the greater the antioxidant activity of BRBE-NEe after in vitro digestion. This is likely due to the increased hydrophobicity of the nanoemulsion, which allows it to better interact with the lipophilic molecules in the digestive environment. These results underscore the significance of the HLB value as a crucial determinant in assessing the antioxidant activity of BRBE-NEe following in vitro digestion. The alterations in phenolic content and antioxidant activity may result from changes in the chemical structure brought about by the process of conjugation during hydrolysis in the intestine by enzymes.^53^ Nemli et al. reported an increase in antioxidant activity of all samples containing tomato pomace emulsions after digestion since the emulsions facilitated the release of antioxidant components from the tomato tissue.^52^

## 4 Conclusions

This research revealed the promising potential of nanoemulsion-based formulations in encapsulating polyphenols extracted from black rice bran. Nanoemulsions could protect the polyphenols from degradation and facilitate their efficient delivery following in vitro digestion while retaining their potent antioxidant activity. Formulating the nanoemulsions with sunflower oil and a mix of hydrophilic and hydrophobic emulsifiers improved the retention of BRBE through hydrophobic interactions. The physicochemical stability analyses showed that HLB significantly affected the nanoemulsions particle size, PDI, ζ-potential, EE, TPC, and antioxidant activity. The HLB 13.25 was the most physically stable formulation, with no significant variation in the mean droplet size, despite demonstrating noticeable changes in TPC and antioxidant activity when stored at 4, 25, and 50 °C over 30 days.

The study also explored the NaCl concentration impact on BRBE-NEe stability, indicating that NaCl concentration significantly affected the nanoemulsions particle size and PDI, and ζ-potential data showed increased negative charge with rising NaCl concentration. Changes in TPC and DPPH values emphasize antioxidant sensitivity to salt concentrations, varying among the HLB formulations.

On the other hand, BRBE-NEe was also stable during in vitro gastrointestinal digestion, and the bioaccessibility of phenolic compounds increased with increasing HLB levels. These results suggest that nanoemulsions can improve the bioaccessibility/bioavailability of polyphenols and other bioactive components. This study contributes to the development of innovative strategies for utilizing nanoemulsion-based formulations as protective carriers for valuable bioactive compounds recovered from food byproducts, with potential applications in functional and fortified foods. Further research is needed to optimize the properties of BRBE-NEe to maximize their potential health benefits.
